# Use of acoustic myography to evaluate forelimb muscle function in retriever dogs carrying different mouth weights

**DOI:** 10.3389/fvets.2022.983386

**Published:** 2022-11-16

**Authors:** Melissa A. Weber, Jane M. Manfredi, Julia E. Tomlinson

**Affiliations:** ^1^Twin Cities Animal Rehabilitation and Sports Medicine Clinic, Burnsville, MN, United States; ^2^Pathobiology and Diagnostic Investigation, College of Veterinary Medicine, Michigan State University, East Lansing, MI, United States

**Keywords:** AMG, tendinopathy, biceps, hunting, myography, weight, mouth, acoustic

## Abstract

**Objectives:**

To evaluate the effect of mouth weight on gait and relative function of forelimb muscles in retriever hunting dogs as a possible explanation for biceps tendinopathy.

**Methods:**

Ten sound retriever dogs underwent acoustic myography, measuring efficiency (E-score), spatial summation (S-score), and temporal summation (T-score) during walk and trot on a pressure-sensitive walkway while carrying a 0 lb (0 kg), 1 lb (0.45 kg), and 3.2 lb (1.45 kg) mouth weight. Gait data included total pressure index (TPI), step length, and stance time. Statistics included a mixed effects model significant at *p* < 0.05.

**Results:**

Forelimb TPI increased with increasing weight. There was no significant change in individual muscle parameters in response to weight. Significance was found in between-muscle comparisons. For walk, T-score was significantly lower in triceps vs. brachiocephalicus with 1 lb, not with 3.2 lb., S-score was significantly lower in the biceps at 0, 1 lb, and triceps at 0 lb. when compared to brachiocephalicus, E-score was significantly lower in deltoideus vs. brachiocephalicus at trot with l and 3.2 lb. There was an overall significant effect of muscle on T-score at trot, but no individual muscle comparison was significant.

**Conclusion:**

Forelimb load increases with mouth weight. Deltoideus had a longer contraction time in response to increasing weight at trot when compared to brachiocephalicus. The biceps muscle did not show increased work in response to increasing weight.

**Clinical relevance:**

The underlying etiology of biceps tendinopathies in retriever dogs remains uncertain but is not due to increasing weight.

## Introduction

Biceps tendinopathies occur in active medium to large breed dogs (1–3). Although no breed predilection has been reported, the clinical experience in a sports medicine specialty practice is that hunting retrievers are overrepresented; hunting retrievers are 25% of the clinic population but 43% of dogs treated for biceps tendinopathy. Biceps tendinopathy in hunting dogs could be due to muscle overuse secondary to increased load on the forelimbs from carrying the weight of a game bird in the mouth–(4–6). The game retrieved can include land birds (Woodcock, Ruffled Grouse and Ring-necked Pheasants) and waterfowl (Canadian Geese, Northern Pintail, and Mallards). The various game can vary in weight from 0.39 lbs (0.18 kg) up to 13 lbs (5.91 kg)^1^^,^ ^2^^,^ ^3^^,^ ^4^.

Previous work by Bockstahler et al. (4) using pressure-sensitive plate analysis showed that peak vertical force and vertical impulse were significantly increased in the forelimbs and not the pelvic limbs when the dogs carried a 0.5, 2, and 4 kg (1.1, 4.4, and 8.8 lb) mouth weight at the walk; and that step length was longer in the forelimbs without a mouth weight as compared to all weights. Gait analysis was not performed at the trot while carrying a mouth weight in the Bockstahler study, which may be important as dogs will cover ground hunting at this gait, but often will gallop or canter to and from a retrieve; and peak vertical force (PVF) is higher at the trot than the walk even though the stance time is shorter (6, 7). The biceps aids in cranial shoulder stabilization during the stance phase of motion and assists in elbow flexion during swing phase (8), the triceps muscle is an anti-gravity muscle that braces the elbow into extension during stance phase and is antagonistic to the biceps and shoulder flexor (8). The deltoideus acts to flex the shoulder joint and plays a minor role as one of the dynamic shoulder joint stabilizers (9). The brachiocephalicus shows low muscle activity during walk and trot (7, 10).

Biceps brachii muscle activity in dogs carrying mouth weights has not been previously evaluated. If the biceps brachii does undergo relative overuse while carrying mouth weights, it could contribute to biceps tendinopathy. Acoustic Myography (AMG) is a validated non-invasive way of assessing muscle function by measuring the sound produced by muscle contractions (11, 12). As muscle fibers contract, they generate vibrations which are recorded by piezoelectric crystals located on transdermal sensors (11, 13). The piezoelectric AMG sensor is thin and minimizes interference from lateral movement on the skin as it only measures sound waves in one direction (11, 13). AMG has been used in dogs in previous studies to evaluate muscle contractions (12–14). The AMG equipment records the sound and calculates three parameters: the E, S, and T-scores with a scale of 0–10. The E-score (efficiency score) reflects coordination of the muscle and muscle activity in relation to inactivity in units of seconds (14). A decrease in E-score while the muscle is working reflects more contraction time vs. relaxation indicating early muscle fatigue (11). The S-score (spatial summation score) reflects signal amplitude as measured in millivolts (mV) (11). A low amplitude during work, indicates that the work is easy, therefore the S-score will be high (13, 14). T-score (temporal summation) is the frequency of muscle fiber recruitment in Hertz (Hz). During very hard work more muscle fibers are recruited, increasing the frequency, resulting in a lower T-score (13, 14).

The objective of this study is to evaluate the effect of mouth weight on gait and function of forelimb muscles in retriever hunting dogs to evaluate them as possible contributors to biceps tendinopathy. We hypothesized that carrying a mouth weight will result in greater recruitment of the biceps brachii, long head of the triceps, and the acromial portion of the deltoideus muscle but not the brachiocephalicus muscle in retriever hunting dogs as measured by AMG, that the muscle activity would increase with increasing weight and that this change would be more pronounced at trot. Secondly, we hypothesized that by carrying mouth weights, the total pressure index (TPI) would be increased in the forelimbs and decreased in the hindlimbs at a walk and trot, and that step length, and stance time would be decreased in the forelimbs when carrying a mouth weight.

## Materials and methods

### Selection criteria

The inclusion criteria to participate in the study were as follows: the dog was a retriever breed, between 2 and 7 years old, between 50 and 80 lbs (22.73–36.36 kg), and free of any previous soft tissue or orthopedic injuries. Dogs were client-owned and written client consent was obtained. The dogs had to be clinically free of lameness as determined by an orthopedic examination, radiographs and gait analysis on a pressure sensitive walkway (gait4dogCIR systems Inc, Franklin, NJ, USA). The dog had to have been active in one or more of the following activities: seasonal waterfowl or upland hunting, hunt tests, field trial, hunting retrieving training, or shed dog hunt. Other sports were also acceptable as long as the dogs met the previously mentioned sport inclusion criteria. The handler also had to believe their dog would be able to work in a heel position holding a mouth weight (dummy) of 1 lb (0.45 kg) and 3.2 lb (1.45 kg) (Real Duck Training Dummy, Moscow ID, USA) at a walk and trot for the duration of the study. Of the 19 dog prospects, 11 dogs passed the inclusion criteria after the dog handler interview. Three dogs were excluded because they had previous orthopedic conditions, two dogs were reported by their handlers to likely not hold the mouth weight (dummy) for the intended time and repetitions, one dog was too fearful and reactive, one dog did not meet the weight criteria, and one dog did not show for the initial appointment.

### Orthopedic evaluation, radiographs, and gait analysis for inclusion into the study

Eleven dogs underwent orthopedic examination performed by a Diplomate of the American College of Veterinary Sports Medicine and Rehabilitation (JET), radiographs of the shoulders and elbows, and gait analysis. The dog's brachial and thigh circumferences were measured using a spring weighted tape measure (Gulick II Warrenville, IL, USA) performed three times per limb with the average measurement being used (15). Each patient underwent goniometry to evaluate passive range of motion of the carpus in flexion, extension, valgus and varus, the elbow in flexion and extension, shoulder abduction angle while the shoulder is in full extension, and shoulder flexion and extension, and a biceps brachii stretch (measured as the degree of elbow extension with the shoulder in flexion and maximal extension of the elbow). Rear limb goniometry was also completed for the hock, stifle, and coxofemoral joints, evaluating passive flexion and extension. Three consecutive goniometric measurements were made for each joint, with the mean value used in accordance with published guidelines (16). If no abnormalities were identified, each dog underwent routine shoulder and elbow radiographs and those with radiographic abnormalities were excluded. One of the 11 dogs did not pass the physical examination as this dog had discomfort on biceps brachii palpation and reduced right biceps brachii stretch. The remaining 10 dogs went on to the final inclusion criteria, the gait analysis.

Gait analysis, using a pressure-sensitive walkway (Gait4dogs, Franklin, NJ, USA), was used to evaluate for lameness. The pressure-sensitive walkway has been previously validated and is calibrated by the manufacturer (17, 18). The dogs were gaited by one handler (MAW). Each dog was familiarized with the environment and walkway with a 10-minute pre-measurement period to acclimate to the room followed by two slow practice walks over the walkway. The dogs were walked and trotted on the pressure-sensitive walkway multiple times in order to obtain three valid passes on the walkway at each gait. A valid pass was recorded if the dog gaited in a straight line, did not step off the pressure-sensitive walkway, and had three gait cycles recorded each pass with a consistent gait (< 10% variability in velocity in a single pass). Real-time video capture of each trial enabled confirmation of straight head position and limb contact. Proprietary designated software (Gait4software^®^ Franklin, NJ, USA) that was made by the same company as the pressure-sensitive walkway was used for acquisition and analysis of the data. A ≤ 6% difference in Total Pressure index (TPI) was accepted as normal between each forelimb and each rear limb during evaluation (19–21). For acceleration during each pass, less than or equal to 10% variability was accepted.

The remaining 10 dogs passed this last inclusion criteria. This time spent during gait analysis provided a sufficient warm up for the dogs before muscle measurements, with an average time of completion of 26 min.

Comparison of the gait parameters of step length and stance time was performed with and without the harness and equipment to rule out any effect of the equipment (shaved hair, AMG sensor, gel, and adhesive) on step length and stance time prior to AMG data collection.

### Data collection

#### Acoustic myography

Prior to data collection, the dogs had previously been acclimated to the location of the pressure-sensitive walkway having completed gait analysis to exclude lameness. The dog was then fitted with a harness (Julius-K9 IDC^®^, Powerharness, Tampa, FL, USA). This harness allowed the AMG recording device (CURO-Diagnostics ApS, Bagsvared, Denmark) to be fixed to the harness under the harness handle. The AMG sensors (MyoDynamik sensors, Copenhagen, Denmark) were 20 mm in diameter and connected to the recording device *via* the designated cables. Two pairs of sensors were run simultaneously, each sensor was placed at the same level on every dog using anatomical landmarks on both the left and right muscle groups.

**Figure 1:**
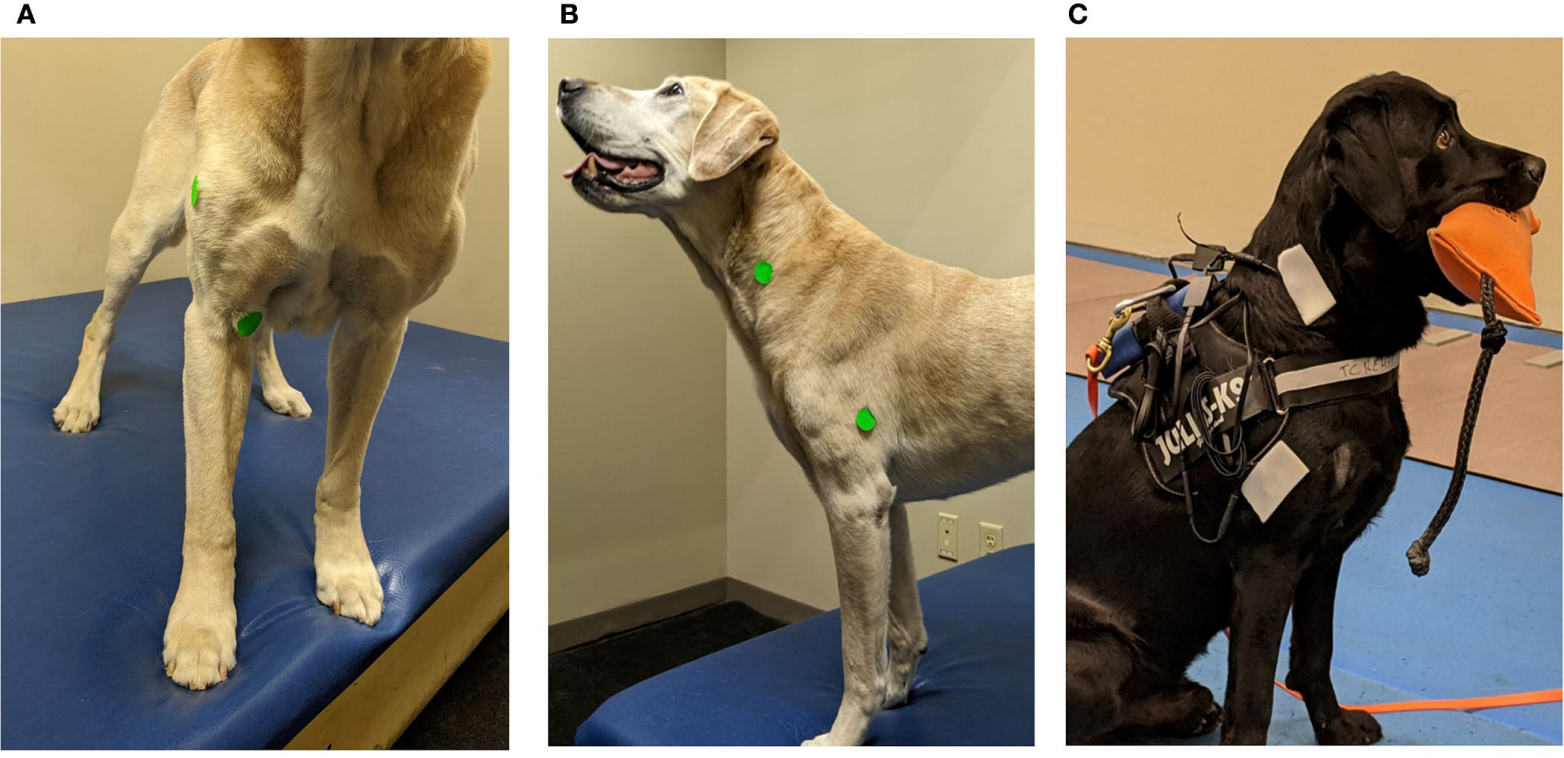
Demonstration of the acoustic myography (AMG) sensor and recording unit placement. **(A)** Green stickers used to demonstrate the placement of sensors in the research dogs. The biceps brachii placed at 2/3 of humeral length and deltoideus acromial portion is placed mid-muscle belly. The dog is a demo dog and not used in the study. **(B)** Green stickers used to demonstrate the placement of sensors in the research dogs. The brachiocephalicus sensor placed at the 4th cervical vertebrae and the triceps long head sensor placed at ½ the length of the humerus. The dog is a demo dog and not used in the study. **(C)** AMG sensor placement with large stickers placed over the small sensors on a research subject. Picture demonstrating equipment set up with sensors placed over the triceps long head and the brachiocephalicus.

The sensor pairing was the biceps brachii and acromial deltoideus muscles, the second muscle pairing was the brachiocephalicus and triceps long head. The sensor pairing order was randomized, each dog proceeded through the gait data collection for each pairing of muscles prior to repeating the data collection with the second muscle pairing. This placement was true for all dogs except one, where the sensor order pairing was different due to error in pairing—sensor pairing was biceps brachii and the long head of the triceps; second pairing was the brachiocephalicus and the deltoideus. For all dogs, the biceps brachii sensor was placed over the palpable muscle belly above the palpable tendon of insertion and below the palpable superficial pectoral muscle at 2/3 humeral length. The deltoideus sensor was centered at the mid-belly of the acromial portion of the deltoideus. For the brachiocephalicus and long head of the triceps, the sensor placement of the brachiocephalicus was at the level of the fourth cervical vertebrae transverse process, and the long head of the triceps, placed over the most caudal muscle belly of the triceps, which can be elevated from the rest of the muscle bellies and was placed at half humeral length (Figure 1).

After the hair at the measurement location was shaved with clippers using a #40 blade, a small amount of acoustic coupling gel (Ekkomarine Medico A/S, Holstebro, Denmark) was placed on the skin and on the sensor. The sensor was placed over the appropriate site and adhered using an adhesive bandage (Snøgg AS, Kristiansand, Norway) placed over the skin and the surrounding coat. The sensor was connected *via* cables to the Smart Sensor slots of the recording device affixed to the harness handle as previously described. The AMG signal from the muscle was transmitted to the recording device then streamed *via* Wi-Fi signal to a hand-held computer tablet (iPad, Apple Inc., Cupertino, CA, USA). The muscle signals could be evaluated and visualized in real time to ensure appropriate transmission of recordings from the sensors.

The dogs were walked and trotted over the pressure-sensitive walkway by the same person (MAW) and gait and muscle data were collected concurrently. The authors chose to rest the dogs between each new set of muscle sensors (average 21.3 min) both to more closely mimic the stop-start of hunting, but also to avoid any possible effects of warm up. This was done in addition to randomizing the order of AMG collection under different weight conditions. On average, the dogs were studied for 4–5 h, with frequent breaks between data sets. The AMG recordings were taken from each of the dogs at a walk and at a trot with no mouth weight, carrying a 1 lb (0.45 kg) mouth weight, and carrying a 3.2 lb (1.45 kg) mouth weight while moving over the walkway. Order of evaluation with weights was randomized. During each weight evaluation, the order of muscle pairs measured was also randomized. Three data recordings for each gait and mouth weight were saved and accepted when the dog walked or trotted across the pressure-sensitive walkway in a straight line holding the mouth weight during the full duration of the walk while the AMG sensors were recording. Dogs could hold the mouth weight anywhere on the body of the mouth weight and were allowed to readjust the bite hold only if it was at the very beginning or very end of the walkway (where data were not recorded) allowing for measurement of three full gait cycles while carrying the weight with no change of bite interruptions. Dogs were not allowed to hold the mouth weight by the string nor were they allowed to drop the mouth weight and pick it back up for the duration of the recorded walk or trot.

The AMG frequency and amplitude were calculated following the protocol by Varcoe et al. (13). During analysis of the AMG muscle data, the threshold was set at 0.2 and adjusted when scores were 0 or 10 (maximum value). Additional set parameters for analysis included a maximum frequency (max T) of 160 Hz (12). This is the maximum firing frequency detectable (22).

#### Gait data collection

Gait data were transmitted from the pressure sensitive walkway to the proprietary designated software for analysis as described above in the inclusion criteria. The dogs were encouraged to keep a steady speed and a straight line across the walkway. Passes were excluded if the dogs stepped off the pressure-sensitive walkway, changed gait, or had an inappropriate acceleration or deceleration (>10% variability in speed). Speed was evaluated within each dog at walk and trot to assess for any variability in speed between passes. Three valid walk and trot data sets consisting of three full gait cycles were analyzed per gait and per mouth weight. Temporospatial parameters and pressure measurements analyzed included total pressure index (TPI), step length (cm), and stance time (seconds). Comparison of the gait parameters of step length and stance time was made prior to AMG data collection with and without the harness and equipment to rule out any effect of the equipment (shaved hair, AMG sensor, gel, and adhesive) on step length and stance time, subsequently gait data used was that collected with the harness and equipment in place.

### Statistical analysis

Normality was determined using a Shapiro-Wilks test. Data were analyzed on a dedicated statistical program (Prism 8, Graph Pad Software, San Diego, CA, USA) using a linear mixed model. The dependent variables were E, S, or T score, independent variables were weight and gait, and muscle, with a random effect of dog. *Post-hoc* tests being Šídák's multiple comparisons test for the AMG data and Tukey's multiple comparison test for the gait data. Gait data at 0 lb mouth weight was also compared with and without wearing AMG equipment. Significance was set at *p* < 0.05.

## Results

### Included dogs

A total of 10 dogs met the inclusion criteria for the study, 8 were Labrador Retrievers, 1 Flat Coat Retriever, and 1 Golden Retriever. Average body weight was 65.04 pounds (29.55 kg) and the average body condition score was 5.3 out of a 9-point system (5 is ideal body condition score) (Nestle PURINA Body Condition System). There were four male intact dogs, three male neutered dogs, two female spayed dogs, and one intact female dog. Each dog was involved in at least one of the following sports: seasonal waterfowl or upland hunting (N = 4), rally obedience (N = 3), hunt test (N = 4), field trial (N = 1), dock diving (N = 1), agility (N = 1), and shed dog hunt (N = 1). One of the dogs participated in four of the listed activities, two dogs participated in two, and the seven other dogs participated in one of the previously mentioned sports. Three of the 10 dogs were not considered to be regularly trained in retrieving (sports-specific fitness) at the time of evaluation as they were practicing retrieves once a week or less (23).

### AMG data

#### Biceps muscle

There was no significant effect of weight on E, S, and T-score at the trot or the walk.

#### Triceps muscle

There was no significant effect of weight on E, S, and T-score at the trot or the walk.

#### Deltoideus muscle

There was no significant effect of weight on E, S, and T-score at the trot or the walk.

#### Brachiocephalicus muscle

There was no significant effect of weight on E, S, and T-score at the trot or the walk.

#### Between muscle comparison

##### E-score

**Figure 2:**
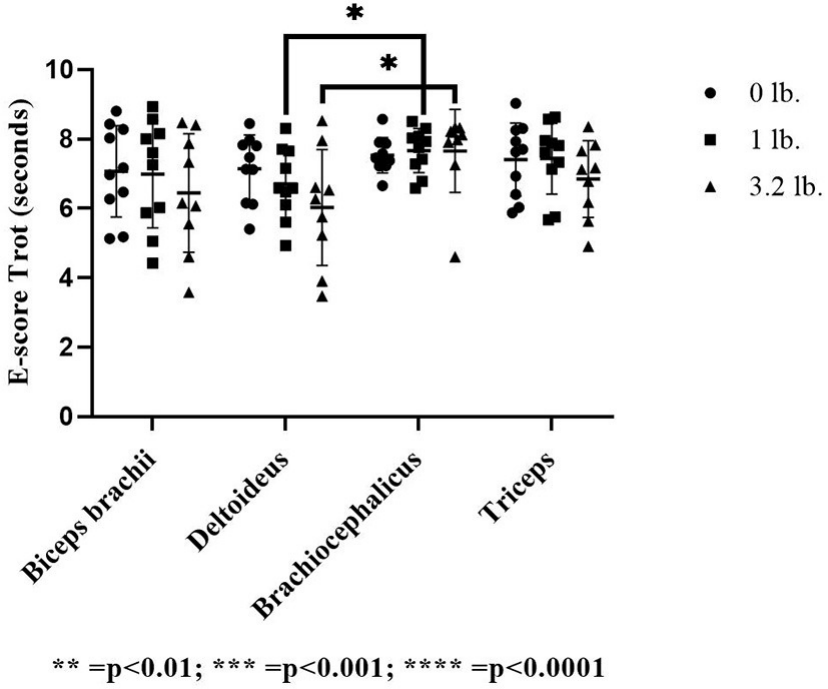
Acoustic myography (AMG) efficiency score (E-score) at trot in the biceps brachii, deltoideus, brachiocephalicus, and triceps (*N* = 10). The asterisks denote different levels of significance. The asterisks denote different levels of significance (*P ≤ 0.05, **P ≤ 0.01, ***P ≤ 0.001, ****P ≤ 0.0001).

At the trot, E-score was significantly lower in the deltoideus at the 3.2 lb (1.45 kg) mouth weight vs. the brachiocephalicus (*p* = 0.03) and the deltoideus vs. brachiocephalicus at the 1 lb (0.45 kg) mouth weight (*p* = 0.04). There was no significant effect between muscle responses to increasing mouth weight at trot for E-score (*p* = 0.78) (Figure 2).

**Figure 3:**
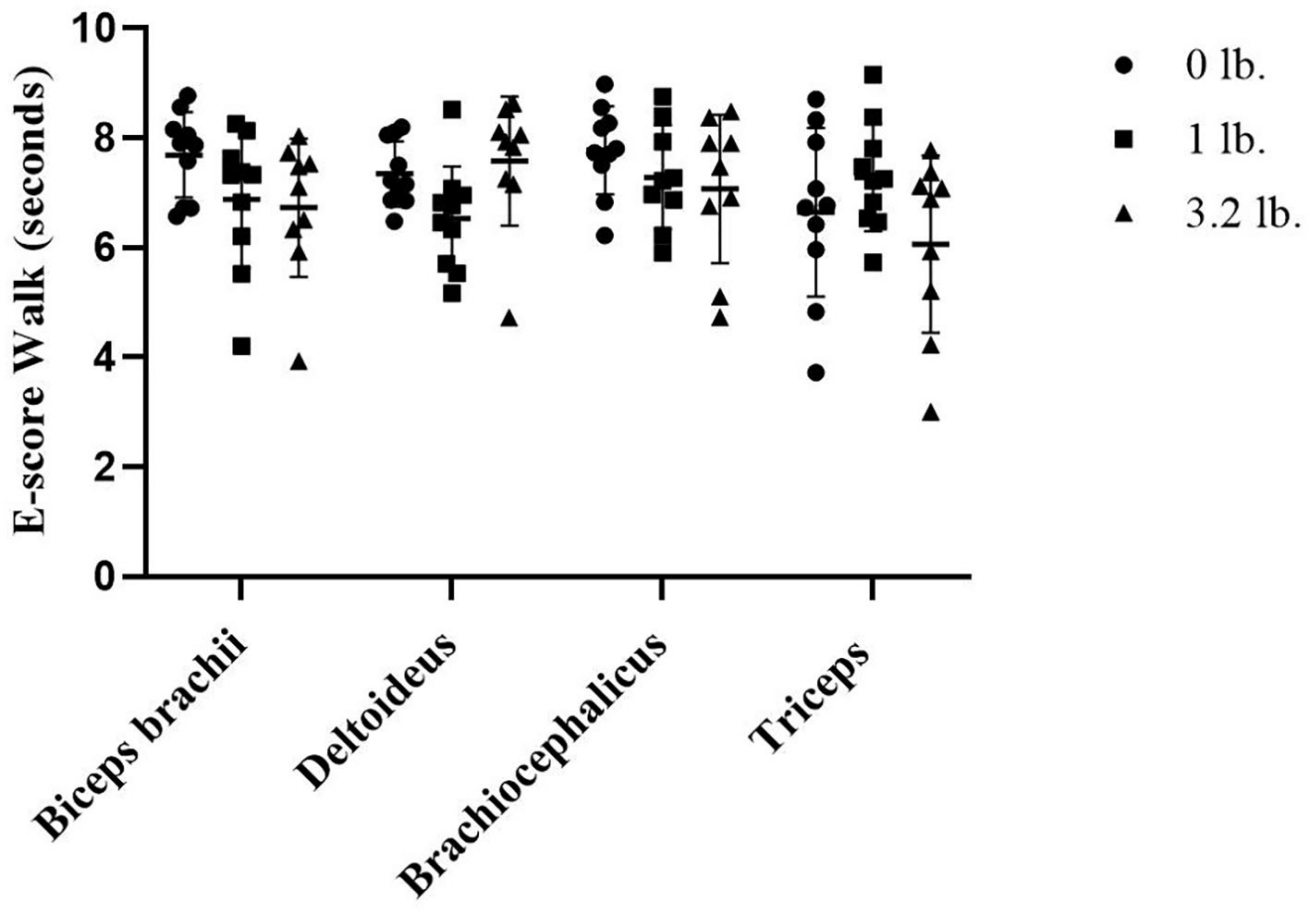
Acoustic myography (AMG) efficiency score (E-score) at walk in the biceps brachii, deltoideus, brachiocephalicus, and triceps (*N* = 10).

At walk, there was a significant effect of muscle-to-mouth weight comparison on E-score (*p* = 0.01) overall, with three of the four muscles (biceps, brachiocephalicus, triceps) decreasing in E-score with increasing weight; however, *post-hoc* testing did not determine pairwise differences likely due to power issues with a conservative *post-hoc* test and a higher number of comparisons. The overall mixed model significance was likely driven by the E-score of the deltoid muscle 0–1 lb (*p* = 0.06), and the triceps 1–3 lbs (*p* = 0.07). There was no significant effect of muscle (*p* = 0.33) or weight (*p* = 0.06) on E-score (Figure 3).

##### S-score

**Figure 4:**
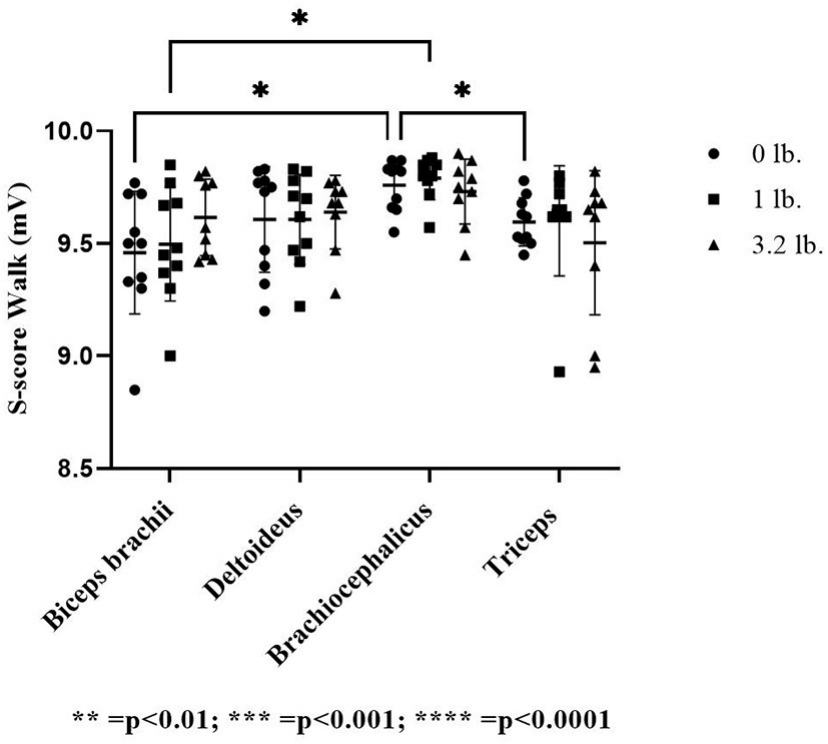
Acoustic myography (AMG) spatial summation score (S-score) at the walk in the biceps brachii, deltoideus, brachiocephalicus, and triceps (*N* = 10). The asterisks denote different levels of significance. The asterisks denote different levels of significance (*P ≤ 0.05, **P ≤ 0.01, ***P ≤ 0.001, ****P ≤ 0.0001).

There was no significant effect of muscle (*p* = 0.22), weight (*p* = 0.19), or between muscles with increasing mouth weight (*p* = 0.59) on S-score at a trot. At walk, the S-score was significantly lower in the biceps (*p* = 0.04) and the triceps (*p* = 0.02) vs. the brachiocephalicus at 0 lb mouth weight. With the addition of 1 lb (0.45 kg) mouth weight, the S-score in the biceps was significantly lower than the brachiocephalicus (*p* = 0.03). There was no significant effect of mouth weight (*p* = 0.85) or between muscle responses to increasing mouth weight for S-score (*p* = 0.38) (Figure 4).

##### T-score

There was a significant effect of muscle on T-score at a trot (*p* ≤ 0.01); however, no individual muscle comparison was significant; comparing the biceps and the brachiocephalicus at the 0 lb vs. the 1 lb (0.45 kg) mouth weight was approaching significance (*p* = 0.05) with the biceps having a lower T-score. There was no significant effect of weight (*p* = 0.17) or between muscle responses to increasing mouth weight for T-score (*p* = 0.61) at the trot.

**Figure 5:**
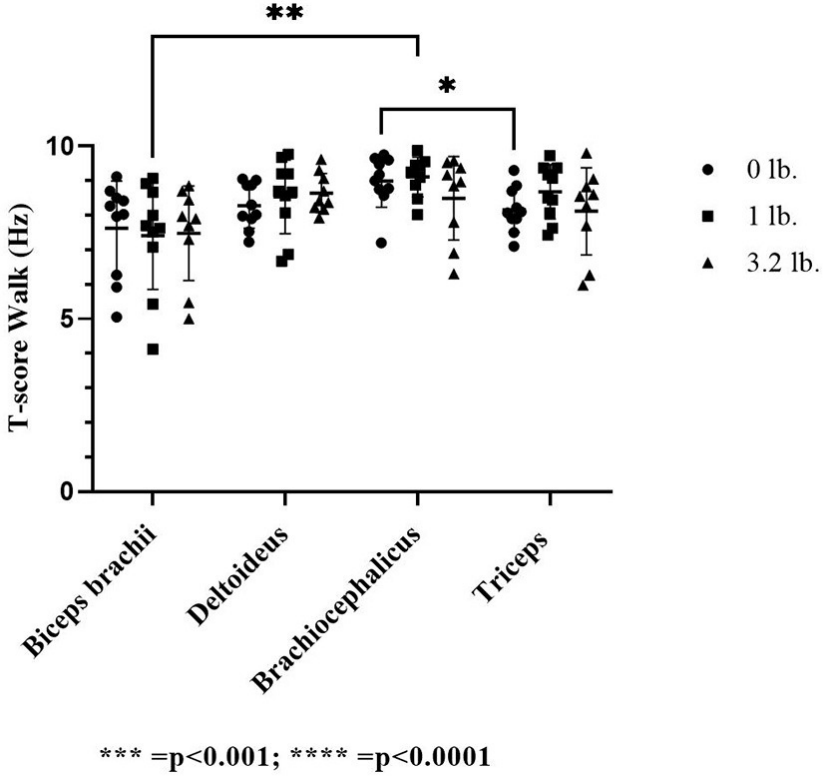
Acoustic myography (AMG) temporal summation score (T-score) at the trot in the biceps brachii, deltoideus, brachiocephalicus, and triceps (*N* = 10). The asterisks denote different levels of significance. The asterisks denote different levels of significance (*P ≤ 0.05, **P ≤ 0.01, ***P ≤ 0.001, ****P ≤ 0.0001).

At the walk, the T-score was significantly lower in the triceps vs. the brachiocephalicus at 0 lb (*p* ≤ 0.05) and in the biceps vs. the brachiocephalicus with the 1 lb (0.45 kg) weight (*p* ≤ 0.01). There was no significant effect of weight (*p* = 0.54) or between muscle responses to increasing mouth weight for T-score (*p* = 0.68) at the walk (Figure 5).

### Gait data

At trot, each individual dog was consistent in speed under different conditions of weight, with a less than 10% variability in speed between all passes on the walkway. At the walk, speed of each individual dog showed more variation, with a maximum of 13% variation in speed between different passes; there was no pattern between weight carried and speed.

There was no significant difference in step length (*p* > 0.27) and stance time (*p* > 0.13) at the walk and trot with and without the harness and equipment, gait data analyzed was that collected with harness and equipment.

#### TPI

**Figure 6:**
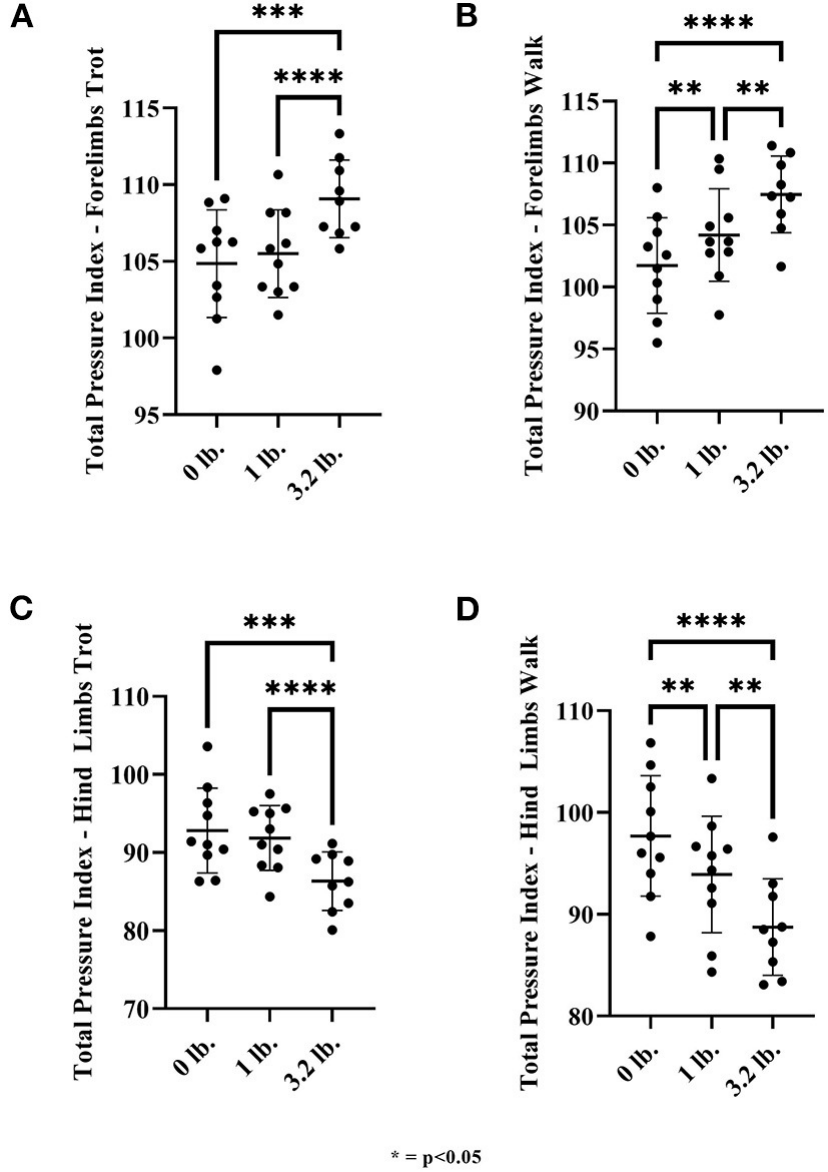
Total pressure index for front and hind limbs at walk and trot. **(A)** Front limb trot, **(B)** Front limb walk, **(C)** Hind limb trot, and **(D)** Hind limb walk (*N* = 10). The asterisks denote different levels of significance (*P ≤ 0.05, **P ≤ 0.01, ***P ≤ 0.001, ****P ≤ 0.0001).

There was a significant effect of mouth weight on TPI in the forelimbs at trot for 0 vs. 3.2 lb (1.45 kg) (*p* < 0.01) and 1 lb (0.45 kg) vs. 3.2 lb (1.45 kg) (*p* < 0.01), with increased total pressure through the forelimbs with the higher weight in each case. There was a significant effect of mouth weight at a walk with 0 vs. 1 lb (0.45 kg) (*p* < 0.01), 0 vs. 3.2 lb (1.45 kg) (*p* < 0.01), and 1 lb (0.45 kg) vs. 3.2 lb (1.45 kg) (*p* < 0.01) with increased total pressure through the forelimbs corresponding to higher weight in all cases (Figure 6). The change in weight between 0 and 1 lb (0.45 kg) at the walk was greater than at the trot between those weights, with a 1.4-fold larger change at the walk vs. the trot [mean TPI changed 2.45 units from 0 to 1 lb (0.45 kg) at walk and 0.65 units from 0 to 1 lb (0.45 kg) at trot]. Similar changes were identified between 0 and 3.2 lb (1.45 kg) with a 1.3-fold larger change at the walk (5.7 units walk, 4.2 units trot).

There was a significant effect of mouth weight at trot with 0 vs. 3.2 lb (*p* < 0.01) and 1 lb (0.45 kg) vs. 3.2 lb (1.45 kg) (*p* < 0.01) on hindlimb TPI at trot, with hindlimb TPI being lower with the higher mouth weight. There was a significant effect of mouth weight at a walk with 0 vs. 1 lb (0.45 kg) (*p* < 0.01), 0 vs. 3.2 lb (1.45 kg) (*p* < 0.01), and 1 lb (0.45 kg) vs. 3.2 lb (1.45 kg) mouth weight (*p* < 0.1) with the hindlimb TPI being lower with increasing weight (Figure 6).

#### Step length

There was no significant effect of mouth weight on step length at the trot in the forelimbs (*p* = 0.23). At walk, there was a significant effect of mouth weight on step length in the 0 vs. 1 lb (0.45 kg) mouth weight (*p* = 0.02), showing an increase in step length with the 1 lb (0.45 kg) mouth weight.

**Figure 7:**
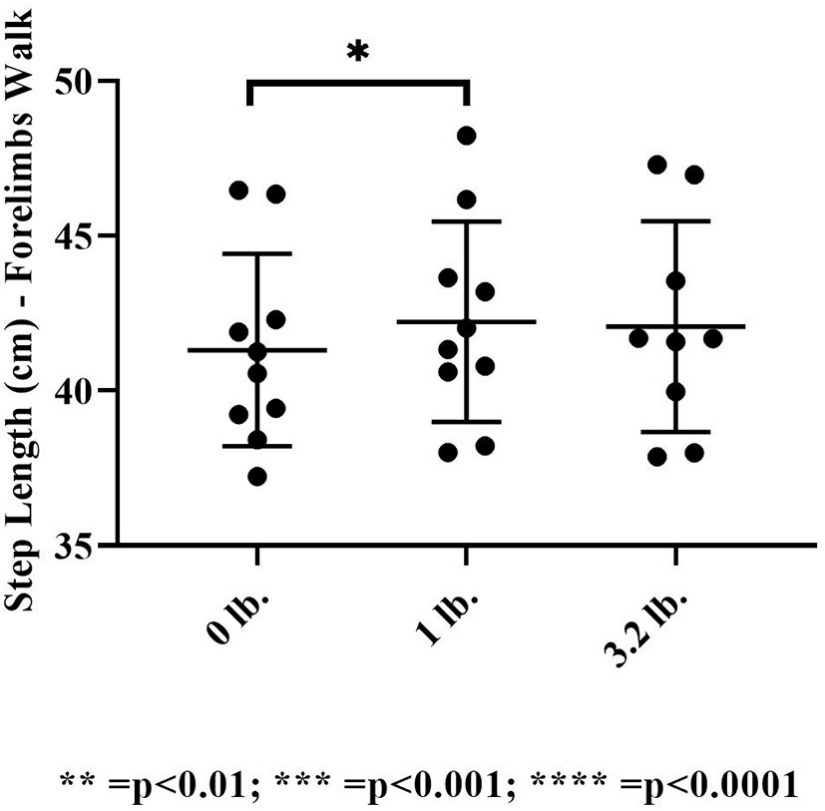
Step length at the walk (*N* = 10). The asterisks denote different levels of significance (*P ≤ 0.05, **P ≤ 0.01, ***P ≤ 0.001, ****P ≤ 0.0001).

There was no significant effect of mouth weight on hind limb step length at the trot (*p* = 0.11) or the walk (*p* = 0.1) (Figure 7).

#### Stance time

There was a significant effect of mouth weight on stance time at the trot in the forelimbs (*p* = 0.03), showing increased stance time with higher weight, but this was not present at the walk (*p* = 0.27). At the trot, there was significantly longer stance time with the 3.2 lb weight than with 0 lb (*p* < 0.01). There was no significance with the 1 vs. 3.2 lb mouth weight (*p* = 0.45), the 0 vs. 1 lb mouth weight was approaching significance with a trend to longer stance time with 1 lb (*p* = 0.05).

**Figure 8:**
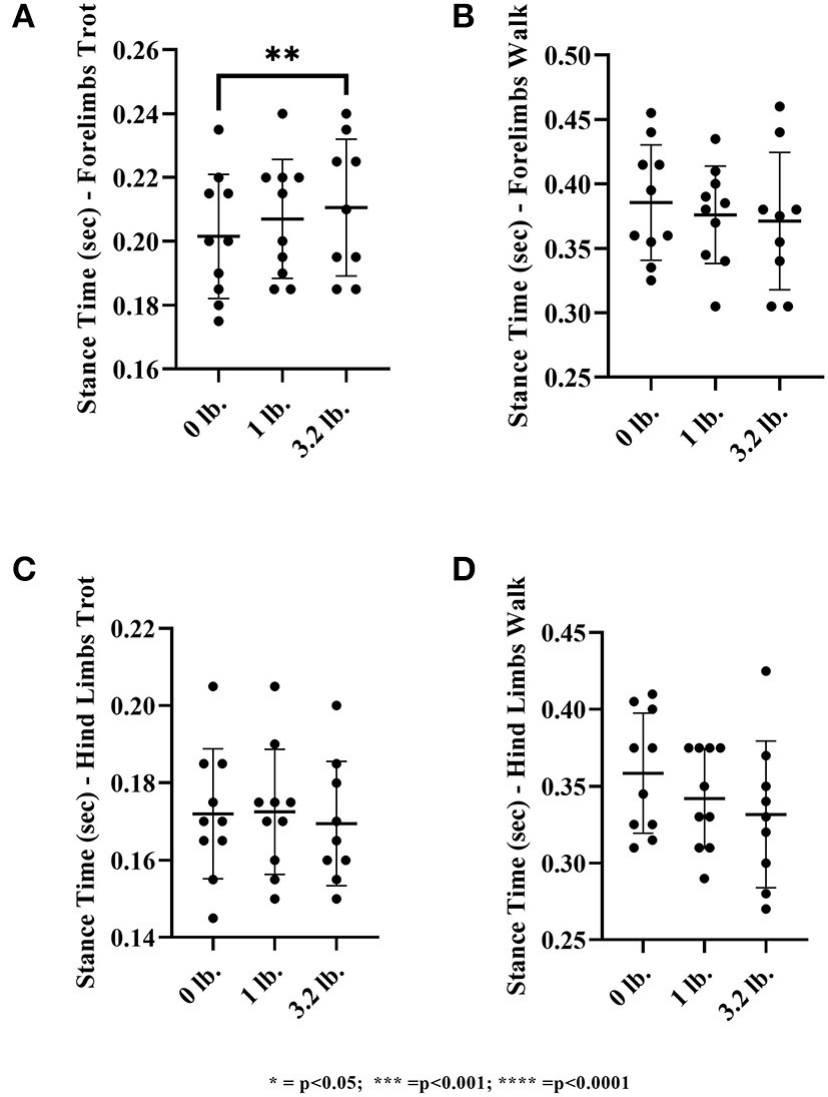
Stance time of the front limb and hind limbs at walk and trot. **(A)** Front limb trot, **(B)** Front limb walk, **(C)** Hind limb trot, **(D)** Hind limb walk (*N* = 10). The asterisks denote different levels of significance (*P ≤ 0.05, **P ≤ 0.01, ***P ≤ 0.001, ****P ≤ 0.0001).

There was no significant effect of mouth weight on stance time at the trot in the hind limbs (*p* = 0.11). There was an overall significant effect of mouth weights at the walk (*p* = 0.03) in the hindlimbs but no significant difference when weight combinations were further evaluated individually (Figure 8).

## Discussion

There was no significant effect of mouth weight within any individual muscle; however, there was a significant effect when evaluating the muscles compared with each other. We compared AMG scores between muscles as well as the changes within a muscle under different conditions of weight because we wanted to see if the relative workload increased in one muscle vs. the others. Uneven workload in the shoulder muscles could be a relative risk factor for muscle injury in the biceps. This choice is not unprecedented as previous studies in people using electromyography (EMG) have compared the activity of different muscles during exercise (24, 25). In this study, the brachiocephalicus showed less action than the other three muscles due to gait and not weight. The function of the deltoideus changed the most in comparison to brachiocephalicus in response to mouth weight; however, it did not show a significant change in comparison to its baseline function. We anticipated that the biceps would have greater muscle fiber recruitment (spatial summation) in response to mouth weight, but it did not. Therefore, we reject our hypothesis that carrying a mouth weight would result in greater recruitment of the biceps brachii, triceps, and deltoideus muscles but not the brachiocephalicus in retriever hunting dogs at walk and trot.

The TPI in the forelimbs at both the walk and trot increased with increasing mouth weight. However, information about stance time and step length was variable. Step length was longer with increasing weight, but only significant at the walk when comparing 0–1 lb (0.45 kg). Stance time was increased but only significant at the trot between 0 and 3.2 lb (1.45 kg). This resulted in our hypothesis that step length and stance time would be decreased in the forelimbs when carrying a mouth weight being rejected.

Similar to Bockstahler et al. (4) pressure-sensitive plate data, the present study found that TPI was significantly increased in the forelimbs and reduced through the hind limbs at the walk with increasing mouth weights. Our significant findings of an increase in step length in the forelimbs at walk between 0 and 1 lb (0.45 kg) differ from the findings of Bockstahler et al. (4), where the forelimb step length was actually longer with no weight compared to all other conditions of mouth weights. There is no clear explanation for this difference in findings. The difference between mean step length at 0 and 1 lb is only 1 cm and so is unlikely to be biologically significant, Bockstahler found a larger (6 cm) mean difference in step length between no weight and the highest weight carried (4 kg). In contrast to our findings of no change in hind limb step length in response to weight, the Bockstahler study (4) found hind limb step length decreased when carrying a 4 kg (8.8 lb) mouth weight; however, the maximum weight we used was 3.2 lb (1.45 kg) which may explain the disagreement for both forelimb and hind limb step length. The weights chosen in the present study were practical for the dogs to carry at trot and correlated to the most common bird sizes retrieved in the United States.

The trot was chosen for this study to help better understand dogs moving at a greater speed, i.e., to cover ground when hunting, recognizing that the gaits have their own biomechanical differences. The lack of change in step length in response to weight at this gait may be because trot is an efficient gait, using energy from elastic storage potential (26) so this could reduce the need for muscle activation; however, a trotting gait has been shown to produce more force through the limbs than a walk (21, 26). A change in muscle function in response to weight carried in the mouth may be more detectable at a canter or gallop, which is the usual natural retrieving gait, but this was not practical for gait analysis.

The main focus of this study, having confirmed increased TPI through the forelimbs in response to mouth weight, was to explore the effect of this increased pressure on muscle function. Overall, the AMG data were supportive of low muscle fiber recruitment, low frequency, and duration of contraction in the brachiocephalicus, both without and with mouth weight. The low muscle activity of the brachiocephalicus (with no weight) is in support of previous studies, which showed that there was low muscle activity of the brachiocephalicus at a constant trotting speed (7) and no difference in function between walk and trot (10) as measured *via* electromyography (EMG). In a study where weight was added to the carpus, (7) brachiocephalicus activity was 313 times greater than baseline, contrasting our findings of mouth weight having no detectable influence on the function of this muscle despite an increase in force through the forelimbs (TPI). Because brachiocephalicus is a forelimb protractor, it is mostly active during early to middle swing phase, though it is active during the last third of stance (8). With the bulk of brachiocephalicus activity being during swing, a weight affixed to the carpus would likely produce resistance to protraction, whereas a mouth weight may not have as much direct effect.

Previous biomechanical studies (8) have found that the biceps brachii tendon of origin is a shoulder stabilizer as part of a shoulder locking mechanism during stance with compression of the supraglenoid tubercle along with tension in the caudal joint capsule, limiting translation (9), the biceps tendon also limits translation of the joint in flexion (27). This constraining action is not dependent on muscular action in the biceps (12, 27). Shoulder flexion may apply tensile stress on the tendon of origin of the biceps, as flexion translates the glenoid cavity caudally in relation to the humeral head (12). The active time period in the biceps muscle as recorded *via* electromyography (EMG) at the trot is only 30% of the gait cycle, vs. 57% at a walk (8). The muscle is most active during walk in the latter two thirds of stance and the first 40% of swing, whereas at trot it is active in the latter half of stance and only 7% of early swing (8). Biceps fiber recruitment (S-score) was significantly greater than brachiocephalicus at the walk without weight (control status), as well as with the 1 lb (0.45 kg) weight, and this was not seen at trot, indicating that this greater fiber recruitment is likely due to longer duration of muscle activation the walking gait. We did find significantly greater frequency of contraction (lower T-score) in the biceps as compared to brachiocephalicus at the walk, but not trot, when carrying 1 lb (0.45 kg), and this could be an effect of carrying mouth weight. At trot, only 26% of the work of locomotion is contracting muscles, the rest being from elastic recoil (26). Knowledge of duration and timing of biceps activity at both gaits and of gait efficiency explains the lack of detectable difference when compared to brachiocephalicus at trot. Even if there was a difference in function of the biceps brachii with increasing weight, it may not be clinically relevant at the trot. At gallop, a gait used when retrieving, 56% of the energy of locomotion was found to be actively shortening muscles, but the hindlimb muscles perform most of that work (26). If the shift in weight distribution found at walk and trot found in the current study holds, then we can expect work to increase in forelimb muscles in response to mouth weight at this gait. However, at the gallop, the biceps brachii is activated for even less of the stride than at trot, at 23% of the total stride time (8); therefore, this could have further challenged our ability to detect a difference in biceps function.

Overall, there was a decrease in the numeric value of E-score at the trot with increasing mouth weights in the biceps, deltoideus and triceps muscles as weight increased, but not in the brachiocephalicus. The deltoideus acts to flex the shoulder joint and plays a minor role as one of the dynamic shoulder joint stabilizers (9). Significantly lower E-score (increased duration of contraction compared to relaxation time in the muscle) was seen in deltoideus at trot when compared to brachiocephalicus at both 1 lb (0.45 kg) and 3.2 lb (1.45 kg). Shoulder flexion is also greater at the trot than at the walk (8) and this is the gait where we found a significant change in deltoideus E-score; however, the greater relative amount of muscle contraction could instead reflect a stabilizing function (9). There is no available previously published myographic data on deltoideus action in the dog, but the muscle should be active in the last half of stance to early swing phase, mirroring published action in the latissimus dorsi which also flexes the shoulder (8). The longer stance time seen in response to mouth weight at the trot should have increased the deltoideus contraction time over the muscle's baseline, which could apply tensile stress on the biceps tendon; however, this was not seen (9).

The triceps muscle is an anti-gravity muscle that braces the elbow into extension during stance phase. We chose the triceps as it is an antagonistic muscle to the biceps (8). There are two main phases of activity of the triceps (8). First, the triceps muscle begins to activate during late swing to touch down. One would think that we would see additional triceps action with increased mouth weight, correlating with increased forelimb TPI but we did not see those AMG changes. The triceps did contract more frequently compared to the brachiocephalicus with no mouth weight. The second phase of triceps contraction begins after a pause in late stance, just before liftoff, ending halfway through swing to help retract the elbow. At trot, the stance phase is shorter and the activation of triceps is shorter. With this efficient gait, this could be why we did not see a change in muscle activity with the triceps in response to weight. The triceps is active for the longest period of time during the gait cycle compared to the biceps and the brachiocephalicus (8) and there is no such data on timing of muscle contraction in the deltoideus.

As more weight (greater TPI) is put through the front limbs with increasing mouth weight, there is a possibility for muscle fatigue in structures not evaluated in this study which could in turn overload the biceps muscle and tendon. Overload of a tendinous structure secondary to fatigue of another structure has been noted in other species to result in tendinopathy (28). This brings the consideration that dogs are becoming fatigued or have inadequate recovery time between events or hunts thus overloading the biceps tendon. Biceps tendinopathies may have nothing to do with biceps muscle contraction but may be due to relative overload, when another structure fatigues.

Potential limitations include a smaller number of dogs evaluated which may have contributed to not reaching statistical significance despite dogs having a lower E-score in the biceps, triceps, and deltoideus muscles with increasing weight. This is pertinent considering the results showed a trend toward a lower E-score at trot in response to increasing mouth weight in the biceps, triceps, and deltoideus muscles, the lack of significant findings may be due to the test being underpowered. Low test power is also a consideration for comparison of the biceps and brachiocephalicus T-score at trot, as the biceps had a numerically lower T-score approaching significance under both 0 and 1 lb (0.45 kg) conditions. Longer stance time with the 1 lb (0.45 kg) vs. 0 lb weight was also approaching significance.

Other limitations include that we did not assess dogs on field surfaces, under prolonged muscle work, and we only evaluated at the walk and trot. The dogs evaluated were all fit, and less fit dogs might be more prone to injury. We were not able to reproduce field conditions, prolonged muscle work was not possible and a walking or trotting gait is not fully reflective of working retriever dogs who normally gallop when carrying a bird. It could be considered another limitation that these dogs were all fit, whereas some dogs may not have a similar level of fitness when carrying birds in the field and it brings to question that the muscle changes in our test subjects may not be fully representative of those dogs prone to biceps injury.

It may have been that muscle function could be altered by warm up as the test progressed for each dog. In the article by Fuglsang-Damgaard et al. (29), they evaluated the triceps muscle activation in agility dogs using AMG during warm up exercises. What they found was the triceps muscle recruited fewer muscle fibers (S-score) and had less duration of contraction (E-score) after warm up. We rested each dog after each pass over the gait mat as that data was being briefly analyzed, dogs were also rested as we transitioned between pairs of AMG sensors. The randomized order of mouth weights should have dealt with the warm up concern, but it may not have been fully avoided.

Not all dogs held the mouth weights in the center and some dogs made bite adjustments; when the bite adjustments were subjectively significant or the position of the mouth weight caused an observably large head tilt, these data sets were excluded. Speed varied between individual passes over the mat for each individual dog and was more variable at walk than at trot, though this variability was a maximum of 13% (due to two dogs the investigators could not get less variable). More than 10% variability in speed at the walk could have affected the force placed on each limb and therefore how the muscle works; however, it was a challenge to get the dog to accept the mouth weight during a walk pass over the mat, which resulted in more variation between passes. Lastly, it is possible that the presence of the harness holding the recording device restricted shoulder motion and therefore affected results. A similar harness to the one used in this study was found to restrict shoulder extension by approximately 2° at walk and by 5° at trot (30). That harness also passed across the chest (though the harness sat directly across the humeri rather than proximal to the humerus as in the case of the harness used in this study). Restriction of shoulder extension could have inhibited brachiocephalicus action; however, the same restriction would be true for all dogs under all experimental circumstances.

The results show that biceps brachii muscle activity did not change significantly in correlation to increased mouth weight. Additional studies are warranted to further evaluate the biceps and additional shoulder muscles in response to mouth weights.

## Data availability statement

The datasets presented in this article are not readily available because data values are included in the graphs within the manuscript. Requests to access the datasets should be directed to drmissy@tcrehab.com.

## Ethics statement

Ethical review and approval was not required for the animal study because the study was non invasive and participants underwent no medical intervention. Written informed consent was obtained from the owners for the participation of their animals in this study.

## Author contributions

MW worked in conjunction with JT in study design, data collection, and analysis. JM contributed to the study design and the grant proposal and assisted MW in running statistical analyses. JT and MW wrote the manuscript with additional input from JM in article editing and proofing. All authors contributed to the article and approved the submitted version.

## Funding

This study was funded by an American Association of Rehabilitation Veterinarians (AARV) and Purina research grant.

## Conflict of interest

The authors declare that the research was conducted in the absence of any commercial or financial relationships that could be construed as a potential conflict of interest.

## Publisher's note

All claims expressed in this article are solely those of the authors and do not necessarily represent those of their affiliated organizations, or those of the publisher, the editors and the reviewers. Any product that may be evaluated in this article, or claim that may be made by its manufacturer, is not guaranteed or endorsed by the publisher.

## Footnotes

^1^ https://www.ducks.org/hunting/waterfowl-id/

^2^ https://www.pheasantsforever.org/Habitat/Pheasant-Facts.aspx

^3^ https://ruffedgrousesociety.org/grouse-facts/

^4^ https://ruffedgrousesociety.org/woodcock-facts/

## Abbreviations

AMG: Acoustic myography
DDF: Deep digital flexor tendon
EMG: Electromyography
E-score: Efficiency score
SDFT: Superficial digital flexor tendon
S-score: Summation score
TPI: Total pressure index
T-score: Temporal summation score.
