# Supplementary figures and images for: Use of acoustic myography to evaluate forelimb muscle function in retriever dogs carrying different mouth weights
Source: Front Vet Sci. 2022 Nov 16;9:983386. doi: 10.3389/fvets.2022.983386 (PMC9709146; doi:10.3389/fvets.2022.983386)

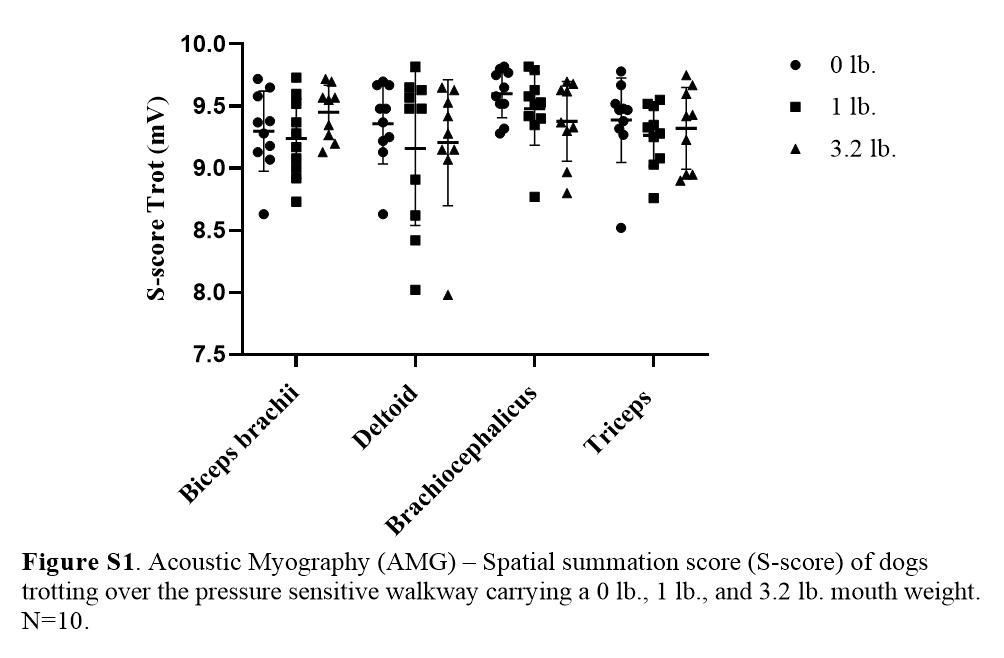

Supplement: Supplementary file 1 [file Image_1.JPEG]

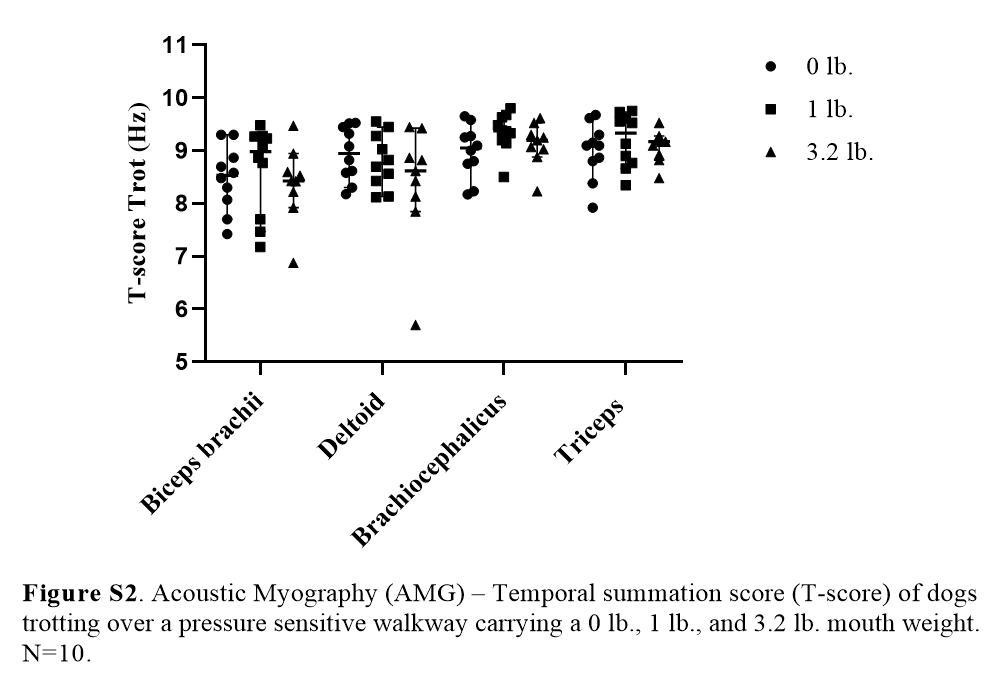

Supplement: Supplementary file 2 [file Image_2.JPEG]

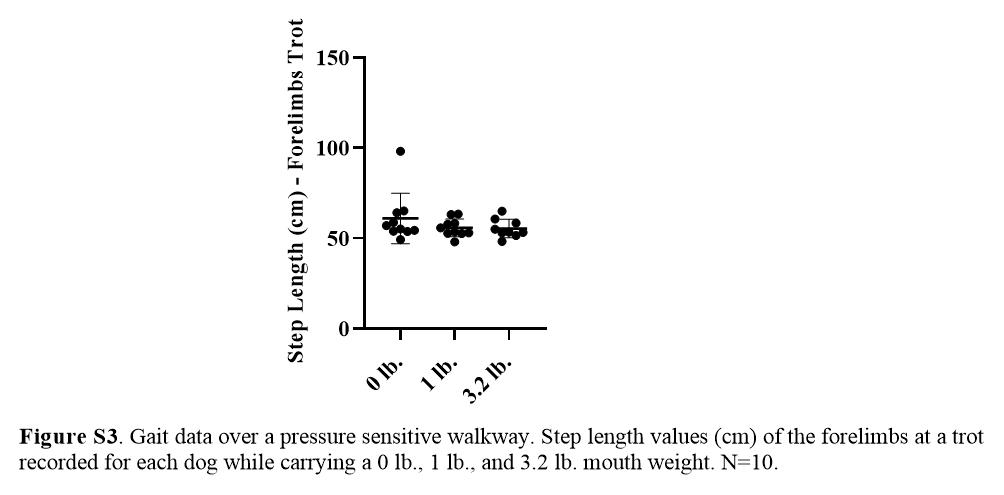

Supplement: Supplementary file 3 [file Image_3.JPEG]

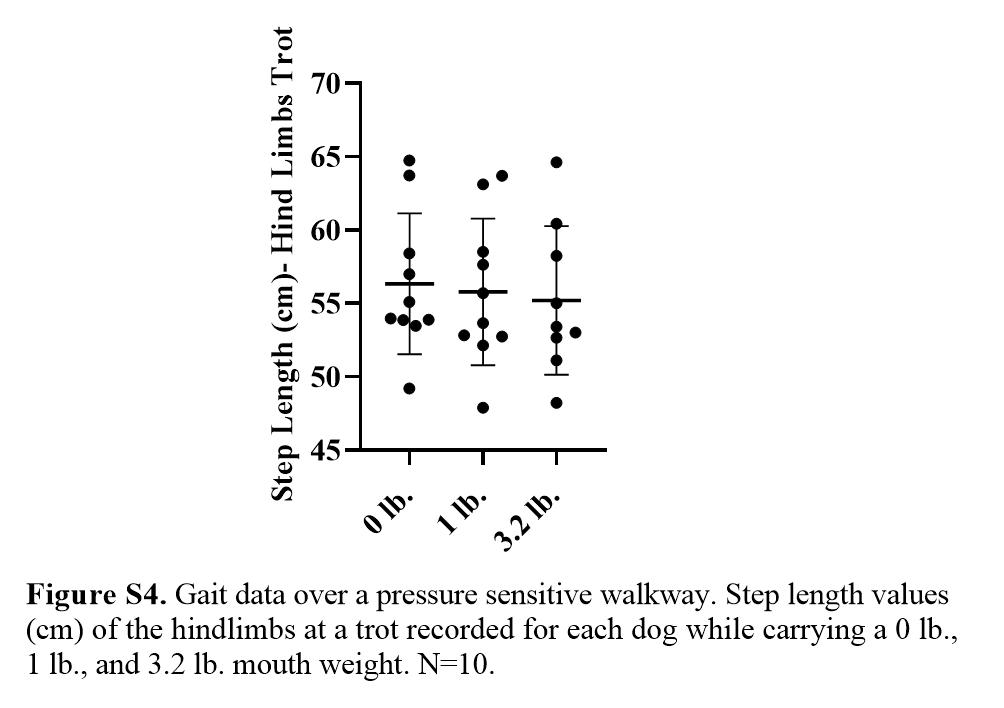

Supplement: Supplementary file 4 [file Image_4.JPEG]

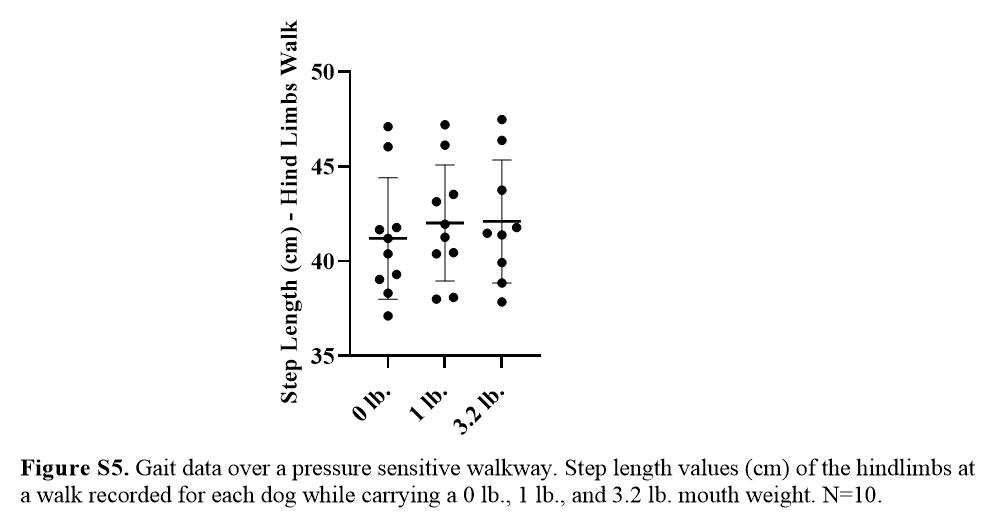

Supplement: Supplementary file 5 [file Image_5.JPEG]
